# A dataset on corporate sustainability disclosure

**DOI:** 10.1038/s41597-023-02093-3

**Published:** 2023-03-31

**Authors:** Jinfang Tian, Qian Cheng, Rui Xue, Yilong Han, Yuli Shan

**Affiliations:** 1 Research Center for Statistics and Interdisciplinary Sciences | School of Statistics and Mathematics, Shandong University of Finance and Economics, Jinan, 250014 China; 2 Centre for Corporate Sustainability and Environmental Finance, Department of Applied Finance, Macquarie University, Sydney, NSW 2109 Australia; 3 School of Economics and Management, Tongji University, Shanghai, 200092 China; 4 School of Geography, Earth and Environmental Sciences, University of Birmingham, Birmingham, B15 2TT UK

**Keywords:** Sustainability, Industry

## Abstract

Enterprises, as key emitters, play a vital role in promoting sustainable development. Corporate sustainability disclosure provides a key channel for stakeholders to gain insights into a company’s sustainability progress. However, few studies have been conducted to measure sustainability disclosure at the firm level. In this study, we apply the machine learning techniques to listed companies’ management discussion and analysis (MD&A) documents and construct a dataset on corporate sustainability disclosure, including the *Corporate Sustainability Disclosure Index* (CSDI), *CSDI_Economic Dimension* (CSDI_ECO), *CSDI_Environmental Dimension* (CSDI_ENV), and *CSDI_Social Dimension* (CSDI_SOCI). The dataset will be updated annually. To the best of our knowledge, this is the first sustainability disclosure dataset constructed at the firm level. Our dataset reflects corporate managements’ sustainability attitudes and promotes the implementation of corporate sustainability strategies and subsequent sustainable economic and social outcomes.

## Background & Summary

The fulfilment of sustainable development goals is a profound issue in today’s economic and social development^1^. Corporate sustainable development helps promote the sustainable development of the economy and the society^2,3^. The disclosure of corporate sustainability related information thus can deliver key practices and performances that firms have contributed to the sustainable development. Accordingly, quantifying corporate sustainable development information can inform shareholders about how firms invest in sustainable transition and provide stakeholders with measurable quantitative benchmarks^4^, motivating firms to make feasible sustainability strategies and take active actions towards sustainable transition.

Existing research on sustainability measurement has largely focused on national and regional levels^5–9^, while research on sustainability at the firm level has remained at the qualitative level^10^. Studies on quantitative measures of corporate sustainability disclosure remain scarce and await empirical investigation.

As stakeholders’ demand for corporate sustainability disclosure increases, more and more international organisations are providing guidelines for corporate sustainability disclosure^11,12^. The Sustainability Reporting Guidelines, developed by the Global Reporting Initiative (GRI), are the most popular among companies worldwide^13,14^. The GRI provides a framework for companies to improve the effectiveness of their sustainability practices^15^. It has also defined the ‘triple bottom line’ (economic, environmental, and social) for corporate sustainability^16^. Thus, constructing a dataset for corporate sustainability disclosure based on the principle of ‘triple bottom line’ can reflect the status of corporate sustainability initiatives in a relatively authoritative manner. The management discussion and analysis (MD&A) part of a company’s annual report reflects the company’s current status and strategic decisions, including sustainability information and strategies^17,18^. As such, quantifying the sustainability-related textual information covered in the MD&A documents can help provide insights into the importance placed by corporate management on sustainability strategies and identify a company’s sustainability capabilities.

China is the world’s largest emerging economy^19^. Meanwhile, it is also the world’s largest carbon emitter^20^ and faces severe hazards such as environmental degradation^21^. Therefore, China has attached great responsibilities to promote sustainable economic and social development^22,23^. Given that firms with superior sustainability performance are more inclined to disclose sustainability information, quantifying Chinese enterprises’ sustainability information disclosure helps reflect and monitor the actual status of their sustainable development. Moreover, it contributes to the timely achievement of the ‘Double Carbon’ strategy^24^.

In this study, we follow the methods of Li *et al*.^25^ about quantifying corporate culture based on latest machine learning techniques and Zhang *et al*.^26^ about constructing a dictionary of environmental characteristics to measure corporate sustainability disclosure. Specifically, we first refer to Zhang *et al*.’s approach to identify sustainability related seed words and then follow Li *et al*.’s approach to process text documents and expand seed words to find their synonyms based on *word2vec* algorithm. Next, consistent with Li *et al*., we apply the *tf.idf* weighting scheme to assign weights (importance) to each word and use the weighted sum of all words to calculate the final corporate sustainability information disclosure index of a specific company at a given year. Taken together, building on the sustainability dictionary constructed using *word2vec*, we build a sustainability disclosure dataset of listed firms in China using the *tf.idf* weighting scheme, including the aggregate *Corporate Sustainability Disclosure Index* (CSDI), *CSDI_Economic Dimension* (CSDI_ECO), *CSDI_Environmental Dimension* (CSDI_ENV), and *CSDI_Social Dimension* (CSDI_SOCI). We also conduct a series of validity tests. First, we test the accuracy of text mining techniques against manual extractions. Second, we examine whether CSDI, CSDI_ECO, CSDI_ENV, and CSDI_SOCI are significantly correlated with the corresponding (real) outcome performance indicators. The results for the validation tests indicate that the constructed corporate sustainability disclosure dataset is valid and reliable, and can help stakeholders track and monitor the actual status of sustainable development of listed firms, which subsequently regulates corporate operations and ultimately promotes sustainable economic and social development.

Our study makes following contributions. First, building on the sustainability framework of ‘triple bottom line’ defined by the GRI guidelines, we employ text mining techniques to construct a corporate sustainability disclosure dataset. To the best of our knowledge, this is the first dataset on firm-level sustainability disclosure measurement. The dataset of corporate sustainable development information disclosure constructed in this study can be applied to investigate the progress and influences of corporate sustainable development, and provide data resources for promoting quantitative research of corporate sustainability^27^, which improves the efficiency of knowledge generation related to sustainable development and saves the social costs spent on related issues. Our dataset can also help entrepreneurs to better design sustainable development strategies at a lower cost and ultimately promote the achievement of global sustainable development^28^.

Second, we mine and quantify sustainability information derived from corporate MD&A documents. MD&A texts reflect the strategic directions and decisions of corporate management, which are closely associated with the company’s sustainability strategies. Thus, our research methodology can be further extended to other texts that reflect executive decisions. For example, at the city level, because government work reports reflect the directions and strategies of government leaders, further studies can examine climate-related textual information in local government work reports to understand local attitudes and initiatives regarding climate governance.

Third, in constructing the ‘Corporate Sustainability Dictionary,’ we expand seed words—words that are closely related to ‘sustainability’—to a larger sustainability dictionary that includes their synonyms based on the *word2vec* technique. This helps avoid the omission of corporate sustainability information in the texts and reduces subjectivity. To more accurately calculate corporate sustainability disclosure indices, we apply the *tf.idf* weight counting scheme, which takes into account the importance of the corporate sustainability-related words in all text corpora^29^. Therefore, the method is able to distinguish between different levels of importance attached to different dimensions of corporate sustainability.

Fourth, the corporate sustainable development information disclosure dataset constructed in this study contributes to the knowledge management literature and expands the application of the serendipity-mindsponge-3D (SM3D) creativity management theory^30^. According to the SM3D framework, innovations are produced through 3-stage information processes: 1) information absorbing and filtering, 2) creativity processing, and 3) innovation outcome. The construction of the corporate sustainability information disclosure dataset follows the SM3D framework and applies the knowledge management theory to sustainable development areas. In the first stage, information on corporate sustainable development were collected from the MD&A documents and irrelevant information were screened out. The information generated from the first stage were then processed using machine learning techniques to create a corporate sustainable development dictionary and weights for each sustainability-related word. The final dataset of corporate sustainable development information disclosure was then produced as an outcome in the last stage. More generally, the SM3D knowledge management framework can be applied to future corporate sustainability management studies.

Last but important, the dataset of corporate sustainable development information disclosure can motivate the private sector to make greater contributions to global sustainable development. As major emission contributors, businesses lack incentives to make green investments willingly as it is hard to quantify their efforts towards sustainable development. The dataset constructed in this study can not only demonstrate the attitudes and strategic direction of corporate management towards sustainable transition but also provide stakeholders such as investors and regulators with quantitative benchmarks. To satisfy stakeholder expectations and so to maintain a positive public image and secure sustained capital flows, corporate executives have a stronger wiliness to make voluntary low-carbon transition. Accordingly, the construction of this dataset can help promote green transition of the private sector and provide solutions to mitigating global sustainability problems such as climate change^31^ and help developing countries to better achieve sustainable development^32^.

## Methods

**Fig. 1:**
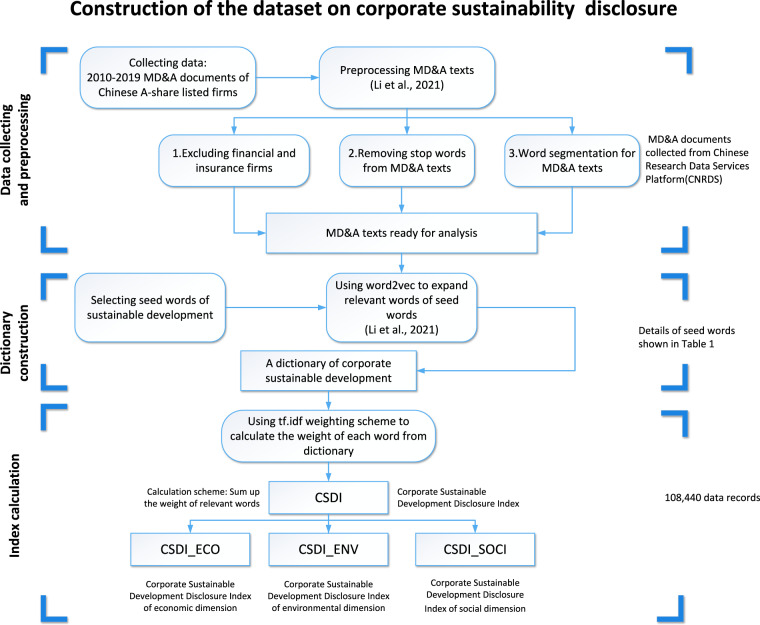
Workflow of the construction of the corporate sustainability disclosure dataset.

The corporate sustainability disclosure dataset is constructed based on companies’ MD&A documents. We collect the data on 29,134 MD&A texts over the period 2010–2019 from the China Research Data Service (CNRDS) platform^33^. The CNRDS database covers sub-databases related to economics, finance and business research, such as economic development section, corporate characteristics section, news and media section, and many more. The MD&A text data used in this study is collected from the listed company text information module of the corporate characteristics section in CNRDS. It is worth mentioning that account registration is needed to log into the CNRDS platform to retrieve and use the data. We exclude financial and insurance companies because the financial sector has adopted different accounting and disclosure rules^34^. Finally, the data on the remaining 27,110 MD&A documents are obtained. Our workflow diagram for constructing the dataset is shown in Fig. 1.

The flowchart presents a brief overview of the process of building the corporate sustainability disclosure dataset. Next, we will explain the process of constructing the corporate sustainability disclosure dataset in detail.

### Pre-processing

To facilitate *word2vec* to ‘read’ the neural network of MD&A texts, ‘learn’ the meaning of the corporate sustainability seed word set, and predict its similarity, we need to first clean the MD&A data and separate the words^35^.

Given that removing stop words improves the accuracy of text mining^36^, we select the following stop word lists that are currently extensively used: the Chinese stop word list, Baidu stop word list, Harbin Institute of Technology stop word list, and Sichuan University stop word list. The removal of stop words in MD&A texts is implemented using Python. Referring to the method of Li *et al*.^25^, we apply the *jieba* word segmentation library in Python to perform word segmentation on the text documents because *jieba* word segmentation technique is widely used in Chinese word segmentation^37,38^. Eventually, the clean MD&A texts of Chinese listed companies from 2010–2019 are obtained following word segmentation.

### The corporate sustainability dictionary

The development of a corporate sustainability dictionary paves the basis for the construction of a corporate sustainability disclosure dataset. This development is achieved through the following two steps: first, we select the corporate sustainability closely-rerated seed words, and second, we expand the seed words to include their synonyms for building a corporate sustainability dictionary. Next, we will explain the process of building a corporate sustainability dictionary in detail.

#### Step 1: Selection of corporate sustainability seed words

We select corporate sustainability seed words based on the three dimensions of economic, environmental, and social, in accordance with the ‘triple bottom line’ defined in the GRI guidelines. To ensure that the set of corporate sustainability seed words is relatively authoritative and convincing, we take the following procedure. First, we extensively check-through the corporate sustainability literature and apply a triangulation process with several experts in the field of corporate sustainability^39^. As a result, the economic dimension of corporate sustainability^40^ includes corporate innovation management^41–44^, risk management^45^, profitability^46,47^, and corporate governance^48^; the environmental dimension covers ecological protection, pollution control, and recycling^49,50^; and the social dimension includes community relations, philanthropy, product quality, information disclosure, and employment relations^51,52^. Second, we finalise corporate sustainability seed words based on the following principles: (1) the selected seed word must appear in the MD&A texts; and (2) after training, the synonyms must complement the meaning of the seed words, following the extension of the *word2vec* model. Finally, a total of 30 corporate sustainability seed words are obtained. Among these, 10 seed words each are dedicated to the economic, environmental, and social dimension, respectively. Table 1 presents the literature sources for the selection of the corporate sustainability seed word set.

**Table 1:** Selection of corporate sustainability seed words for each dimension.

Corporate sustainability dimension	Subdimension	Seed words	Sources
Economic dimension	Innovation management	Innovation	Guiso *et al*.^52^
		Efficiency	Nini *et al*.^79^
	Risk management	Repayment	
		Risks	Power (2009)^45^
	Profitability	Profit	Grunig (1979)^80^
	Corporate governance	Performance	Nini *et al*.^79^
		Growth	
		Development	
		Expenses	
		Management	
Environmental dimension	Ecology and environmental protection	Ecology	Sharma & Henriques (2005)^49^
		Climate	
		Green	Prasad & Elmes (2005)^81^
		Environmental protection	
	Pollution control	Emission reduction	Sharma & Henriques (2005)^49^; Chan(2005)^82^
		Pollution	
	Recycling	Low carbon	Sharma & Henriques (2005)^49^; Chan(2005)^82^
		Waste	Bansal (2005)^1^; Chan (2005)^82^
		Energy saving	Sharma & Henriques (2005)^49^; Chan(2005)^82^
		Renewable	Bansal (2005)^1^; Chan (2005)^82^
Social dimension	Community relations	Community	Guiso *et al*.^52^
		Caring	
	Philanthropy	Ethics	Cochran & Wood (1984)^51^
		Donation	
	Product quality	Safety	
		Responsibility	
	Information disclosure	Transparency	Bansal (2005)^1^; Chan (2005)^82^
		Fairness	
	Employment relations	Welfare	Sonenshein (2016)^83^
		Team	Guiso *et al*.^52^

#### Step 2: Generation of corporate sustainability dictionary

As a latest machine learning technique in natural language processing (NLP) and an open-source tool to produce word vectors^53^, *word2vec* uses neural networks to more accurately learn low-dimensional vectors that represent word meanings, converts words into vector representations, and predicts similarity between words based on the *cosine* similarity method^54^. In this study, we extend seed words and find their synonyms through using *word2vec* to obtain the semantic similarity between words in the MD&A texts. Accordingly, we use the trained *word2vec* model to extend the seed words to include their synonyms based on the full MD&A documents to construct the corporate sustainability dictionary.

To facilitate understanding, we take the extension of the synonyms in the economic dimension as an example. First, we assume that the MD&A corpus contains V words, and the initial word vector dimension of each seed word is *V*. The *word2vec* model reduces the dimension of each word vector to ensure that the word vector dimension is set in such a way that it can summarise the meaning of the seed words in a more comprehensive way without being excessively redundant. Following Li *et al*.’s^25^ method of reducing the dimension of the word vector of each corporate cultural seed word, we set the word vector dimension to 300. Then, we have the word vector of ‘innovation’ is ${V}^{\{1\}}=[{x}_{1}{}^{\{1\}},{x}_{2}{}^{\{1\}},{x}_{3}{}^{\{1\}},\ldots {x}_{300}{}^{\{1\}}]$, word vector of ‘efficiency’ is ${V}^{\{2\}}=[{x}_{1}{}^{\{2\}},{x}_{2}{}^{\{2\}},{x}_{3}{}^{\{2\}},\ldots {x}_{300}{}^{\{2\}}]$, and the word vector of ‘management’ is ${V}^{\{10\}}=[{x}_{1}{}^{\{10\}},{x}_{2}{}^{\{10\}},{x}_{3}{}^{\{10\}},\ldots {x}_{300}{}^{\{10\}}]$. Here, ${x}_{i}^{\{j\}}$ denotes the dimension *i* of the *j*th seed word. Next, we calculate the similarity between the word vectors of each word in the MD&A corpus and the ten seed words. We follow the approach of Li *et al*.^25^ in calculating the similarity between words using the *cosine* similarity between word vectors. The *cosine* similarity is expressed as the *cosine* of the angle between two word vectors $A=({a}_{1},{a}_{2},\ldots {a}_{n})$ and $B=({b}_{1},{b}_{2},\ldots ,{b}_{n})$, following the formula shown below in Eq. (1). The higher the *cosine* similarity, the closer the *cosine* value of the angle between the two word vectors is to 1. This indicates that the more the angle between the two word vectors converges to 0, the more similar the two words are.

1 $${\rm{sim}}(A,B)=\frac{A\cdot \,B}{| | A| | \times | | B| | }$$

To ensure a sufficient number of synonyms, we first select 30 synonym words with the highest *cosine* similarity to each seed word. After obtaining the 30 synonyms, we manually check-through all of them to exclude words with inappropriate or irrelevant meaning to sustainability. As a last screening step, for each seed word, we retain top 10 synonyms with the highest *cosine* similarity to the vector of the seed word. Following the same approach, we obtain 100 words each for the environmental and social dimensions of corporate sustainability, respectively. Ultimately, we construct a corporate sustainability dictionary comprising a total of 330 words (30 seed words + 30*10 synonyms of the seed words).

### Construction of the corporate sustainability disclosure dataset

In this sub-section, we explain the process of constructing the Corporate Sustainability Disclosure Dataset (CSDI, CSDI_ECO, CSDI_ENV, CSDI_SOCI) in two steps.

#### Step 1: Weighting scheme

Referring to Li *et al*.’s^25^ method of quantifying corporate culture, we utilise the *tf.idf* weighting scheme to calculate the weight of each word. This weighting scheme takes into account the importance of corporate sustainability-related words in a particular company’s MD&A document and the entire MD&A corpus. Specifically, in this study, *tf* (Term Frequency) denotes the frequency of a word in a company’s MD&A for a given year. Considering the word ‘innovation’ as an example, its frequency *tf* in text *j* is:

2 $${tf}_{inovation,j}=\frac{the\;frequency\;of\;the\;word\;{\prime\prime} inovation{\prime\prime} \;inMD\& A\;texts}{Total\;word\;counts\;of\;this\,MD\& A\;text}$$

*idf* (inverse document frequency) denotes the inverse document frequency of a word in the texts of the MD&A corpus. In simple terms, *idf* represents the general importance of a word in the MD&A corpus. Considering the example of ‘innovation’ again, the inverse document frequency of ‘innovation’ in the MD&A corpus is:

3 $$id{f}_{innovation}={\rm{\log }}\left(\frac{Total\;number\;of\;MD\& A\;texts\;in\;the\;corpus}{Number\;of\;texts\;containing\;{\prime\prime} innovation{\prime\prime} +1}\right)$$

Finally, we obtain the weight of ‘innovation’ in text *j* as follows:

4 $$tfid{f}_{innovation,j}=t{f}_{innovation,j}\times id{f}_{innovation}$$

We apply the *tf.idf* weighting scheme to calculate the weight of each word in the corporate sustainability dictionary.

#### Step 2: Calculation of the corporate sustainability disclosure index (CSDI)

We then use the weighted sum of all words’ frequency in the corresponding sustainability dictionary to derive the CSDI at the firm-year level. We also apply the same methodology to each of the three dimensions to derive the *CSDI_Economic Dimension* (CSDI_ECO), *CSDI_Environmental Dimension* (CSDI_ENV), and *CSDI_Social Dimension* (CSDI_SOCI). Accordingly, the corporate sustainability disclosure dataset (CSDI, CSDI_ECO, CSDI_ENV, CSDI_SOCI) is generated.

### Statistical analysis of the corporate sustainability disclosure dataset

In this section, we report summary statistics, summarise the trend of each dimension index, and analyse the correlation between each dimension index to provide a preliminary understanding of the corporate sustainability disclosure dataset. The results are presented in Table 2.

**Table 2:** Descriptive statistics.

Panel A – Summary statistics				
Variable	Mean	Std. Dev.	Min	Max
CSDI	0.233	0.122	0.000	1.447
CSDI_ECO	0.130	0.052	0.000	1.124
CSDI_ENV	0.063	0.095	0.000	1.365
CSDI_SOCI	0.041	0.043	0.000	0.528
Panel B – Autocorrelations of CSDI (CSDI_ECO, CSDI_ENV, CSDI_SOCI)				
Variables in year t	Year t-1	Year t-2	Year t-3	Year t-4
CSDI	0.107***	0.110***	0.080***	0.075***
CSDI_ECO	0.085***	0.100***	0.084***	0.072***
CSDI_ENV	0.058***	0.055***	0.037***	0.019***
CSDI_SOCI	0.181***	0.172***	0.165***	0.175***
Panel C – Correlations of CSDI, CSDI_ECO, CSDI_ENV, CSDI_SOCI				
Variables	CSDI	CSDI_ECO	CSDI_ENV	CSDI_SOCI
CSDI	1.000			
CSDI_ECO	0.479***	1.000		
CSDI_ENV	0.810***	0.017**	1.000	
CSDI_SOCI	0.490***	0.119***	0.114***	1.000

Notes: Panel A lists the descriptive statistics of CSDI, CSDI_ECO, CSDI_ENV, CSDI_SOCI; Panel B presents the changing trends of CSDI, CSDI_ECO, CSDI_ENV, CSDI_SOCI; Panel C shows the correlations between CSDI and CSDI_ECO, CSDI_ENV, CSDI_SOCI; and ***p < 0.01, **p < 0.05, *p < 0.1.

First, Table 2 Panel A presents the descriptive statistics for the corporate sustainability disclosure dataset. The mean value of economic dimension (CSDI_ECO) is the largest, suggesting that economic sustainability is the basis for corporate sustainable development^55^. Moreover, the mean value of social dimension (CSDI_SOCI) is the smallest, which provides evidence to support the statement of Wartick and Cochran^47^ that corporate social responsibility is less important than the maximisation of corporate profits. Furthermore, the variance of environmental dimension (CSDI_ENV) is the largest, indicating that a significant difference exists in the degree of importance placed on environmental protection by different corporate executives. This finding also implies that promoting corporate environmental management is indispensable in the process of advancing sustainable economic and social development.

Second, we analyse the patterns in the corporate sustainability disclosure dataset. We investigate the consistency of CSDI, CSDI_ECO, CSDI_ENV, and CSDI_SOCI over the five-year window and display the correlation coefficients of each dimension of the corporate sustainability disclosure index for the current period with its lagged periods in Panel B of Table 2. We find that the correlation coefficients between current year and years *t-1* to *t-*4 are all significantly positive, suggesting that a consistent attitude of corporate management towards sustainability.

Third, we examine the correlation between CSDI and each dimension index. Table 2 Panel C presents the correlations between CSDI and CSDI_ECO, CSDI_ENV, and CSDI_SOCI. The results indicate that CSDI is significantly and positively correlated with CSDI_ECO, CSDI_ENV, and CSDI_SOCI, and there also exists a significant positive correlation between CSDI_ECO, CSDI_ENV, and CSDI_SOCI. This corroborates the fact that in the long run, enterprises undertaking environmental and social responsibilities will help contribute to the maximisation of the company value; that is, corporate environmental and social performance will translate into financial performance^56^.

## Data Records

We upload a total of 108,440 data points from the Corporate Sustainability Disclosure dataset. Besides, the corporate sustainability dictionary is also uploaded. The data records consist of the following datasets:

- The Corporate Sustainability Disclosure Dataset (CSDI, CSDI_ECO, CSDI_ENV, CSDI_SOCI) contains a total of 108,440 (27,110*4) data points^57^. Notably, this dataset has an individual file for each year (2010–2019) with column headings of “Year” describing the year of the dataset, “Corporate Code” and “Corporate Name” representing the name and stock code of the company, “CSDI” displaying the overall disclosure index of corporate sustainable development, “CSDI_ECO”, “CSDI_ENV”, and “CSDI_SOCI” listing the respective dimension disclosure index, and “Industry Code” indicating the industry classification code of the company.
- The Corporate Sustainability Dictionary includes a total of 330 words (30 seed words + 30*10 synonyms of the seed words)^58^.
- The data used in the Technical Validation section includes corporate indicators of economic performance, environmental performance, and social performance, as well as a battery of control variables^59^.

## Technical Validation

In this section, we examine the accuracy of the text mining techniques used to construct the dataset and validate the relationship between the corporate sustainability disclosure dataset and corresponding corporate actual performance.

### Validation of the text mining techniques

To verify the accuracy of the text mining technique employed in this study, we randomly select ten MD&A documents from different industries across different years, and manually retrieve ten words included in the constructed corporate sustainability dictionary. The differences between the manual check and text mining results are compared and reported in Table 3. Panel A of Table 3 presents the number of occurrences of sustainability-related words in the corresponding MD&A documents using the manual retrieval method, while Panel B presents the corresponding results based on the text mining techniques. It is clear that the results of word counts based on manual retrieval and the text mining techniques are identical.

**Table 3:** Manual-checking for sample text mining results.

Panel A – Manual Search Word Count										
	Chemical fibre manufacturing	Air transport industry	Capital markets services	General equipment manufacturing	Catering	Road transport industry	Wholesale trade	Rubber and plastic products industry	Real estate	Computer, communications, & other electronic equipment manufacturing
Energy saving	0	0	4	16	1	0	0	0	0	0
Profit	6	6	5	0	0	3	1	3	4	9
Responsibility	0	7	3	2	3	0	2	0	0	0
Efficiency	0	1	1	1	0	0	0	0	3	4
Environmental protection	1	1	7	10	1	7	7	0	0	0
Safety	0	37	3	3	5	10	5	2	1	0
Development	9	28	36	23	32	27	8	36	27	14
Ecology	0	0	0	0	0	0	0	0	0	0
Fairness	0	0	0	0	0	0	0	0	1	0
**Panel B – Text Mining Word Count**										
Energy saving	0	0	4	16	1	0	0	0	0	0
Profit	6	6	5	0	0	3	1	3	4	9
Responsibility	0	7	3	2	3	0	2	0	0	0
Efficiency	0	1	1	1	0	0	0	0	3	4
Environmental protection	1	1	7	10	1	7	7	0	0	0
Safety	0	37	3	3	5	10	5	2	1	0
Development	9	28	36	23	32	27	8	36	27	14
Ecology	0	0	0	0	0	0	0	0	0	0
Fairness	0	0	0	0	0	0	0	0	1	0

Notes: Panel A reports the word counts under manual retrieval; and Panel B lists the word counts based on text mining techniques.

### Validation of the corporate sustainability disclosure index

To further ascertain the validity of the corporate sustainability disclosure dataset, we follow the approach of Li *et al*. to examine the effectiveness of the disclosure indexes^25^. For the purposes of this study, we collect corporate real performance indicators for each of the three sustainability dimensions. To control for the potential effects of other factors on the regression results, we add a battery of firm-level control variables^60–63^ when examining the relationship between each dimension disclosure index and the corresponding corporate real sustainability performance in that dimension. The following firm-level control variables are added: financial leverage (*leverage*), return on assets (*ROA*), shareholding of the largest shareholder (*Share1*), whether audited by a Big Four auditor company (*Big4*), firm size (*Size*), and firm age (*Listage*). The above data are all obtained from the CSMAR database^64^ and are available when logged in. The validation results are described in detail below.

First, we test the validity of *Corporate Sustainability Disclosure Index_Economic Dimension* (CSDI_ECO). To verify the validity of CSDI_ECO, we examine the relationship between CSDI_ECO and the following corporate economic performance indicators: total factor productivity (*TFP*), growth in revenue (*Growth*), bankruptcy risk (*O’score*), and financial constraints (*SA*). First, TFP reflects the efficiency of converting inputs into outputs in the production process. Thus, at the firm level, a firm’s TFP can represent the firm’s ability to become economically sustainable. We calculate TFP based on the method developed by Levinsohn and Petrin^65^. Second, we use revenue growth to reflect the economic growth ability of the firm^66^. Third, because financial constraints limit firms’ investment in research and development (R&D) activities^67^, which will affect firms’ long-term economic development, we select the *SA* index^68^ to represent firms’ actual financial constraints. The larger the *SA* index, the more severe the firms’ financial constraints are, and so the less sustainable their economic development. Lastly, we select *O’score*^69^ to reflect corporate economic sustainability from the perspective of risk management, with a larger *O’score* representing greater distress in business operations and so higher likelihood of being bankruptcy. The validation results are reported in Table 4. Overall, the associations of CSDI_ECO with all of the four economic indicators are highly correlated. Thus, the validation results shown in Table 4 provide solid evidence that the CSDI_ECO constructed in this study can reflect the actual performance of firms’ economic sustainability.

**Table 4:** Validation of CSDI_ECO.

VARIABLES	*TFP*	*Growth*	*SA*	*O’score*
*CSDI_ECO*	0.384***	0.145***	−0.203***	−2.832***
	(3.87)	(2.83)	(−5.31)	(−4.83)
*Leverage*	0.930***	−0.033**	−0.174***	5.947***
	(28.14)	(−1.98)	(−14.54)	(32.31)
*ROA*	3.281***	0.538***	−0.020	−4.017***
	(33.11)	(10.38)	(−0.56)	(−6.54)
*Share1*	0.195***	0.007	0.193***	2.428***
	(6.51)	(0.38)	(14.73)	(12.25)
*Big4*	0.093***	0.025**	0.096***	0.563***
	(4.81)	(2.47)	(9.33)	(5.37)
*Size*	0.641***	0.004	0.008***	−1.118***
	(123.30)	(1.40)	(3.29)	(−39.68)
*Listage*	0.009	0.007*	−0.008***	−0.023
	(1.26)	(1.77)	(−2.58)	(−0.51)
*Constant*	−6.025***	−0.436***	−3.662***	14.408***
	(−50.98)	(−6.76)	(−72.36)	(22.45)
*Observations*	13,758	15,599	15,599	15,599
*R-squared*	0.800	0.183	0.253	0.187
*Industry Fixed Effects*	YES	YES	YES	YES
*Year Fixed Effects*	YES	YES	YES	YES

Notes: ***p < 0.01, **p < 0.05, *p < 0.1; robust t-statistics in parentheses.

Second, we examine the validity of *Corporate Sustainability Disclosure Index_Environmental Dimension* (CSDI_ENV). To verify the validity of the CSDI_ENV, we test the relationship between CSDI_ENV and corporate environmental performance indicators. The following indicators are selected: disclosure of dust control (*Dust_control*), disclosure of wastewater discharge (*Wastewater*), disclosure of solid wastes utilisation and disposal (*Solid_waste*), and disclosure of waste gas abatement and control (*Waste_gas*)^70^. The validation results are presented in Table 5. The associations between CSDI_ENV and all of the four corporate environmental indicators are highly correlated. The results document that CSDI_ENV constructed in this study is positively associated with companies’ environmental performance.

**Table 5:** Validation of CSDI_ENV.

VARIABLES	*Dust_control*	*Wastewater*	*Solid_waste*	*Waste_gas*
*CSDI_ENV*	2.886***	3.144***	3.081***	3.781***
	(10.96)	(12.57)	(12.49)	(14.51)
*Leverage*	−0.159	−0.347**	−0.539***	−0.242*
	(−0.94)	(−2.52)	(−3.65)	(−1.70)
*ROA*	−1.197**	−0.013	0.783*	−0.448
	(−2.42)	(−0.03)	(1.85)	(−1.10)
*Share1*	0.417**	0.103	−0.142	0.214
	(2.47)	(0.73)	(−0.96)	(1.47)
*Big4*	0.281***	0.324***	0.393***	0.583***
	(2.96)	(3.69)	(4.56)	(6.67)
*Size*	0.506***	0.483***	0.520***	0.528***
	(19.63)	(21.42)	(22.18)	(22.78)
*Listage*	−0.121***	−0.049	−0.116***	−0.079**
	(−3.22)	(−1.54)	(−3.56)	(−2.47)
*Constant*	−13.908***	−13.154***	−14.659***	−14.438***
	(−21.06)	(−23.66)	(−21.62)	(−25.20)
*Observations*	15,139	15,529	15,501	15,516
*Industry Fixed Effects*	YES	YES	YES	YES
*Year Fixed Effects*	YES	YES	YES	YES

Notes: ***: *p* < 0.01, **: p < 0.05, *: p < 0.1; robust t-statistics in parentheses.

Third, we provide the validation of *Corporate Sustainability Disclosure Index_Social Dimension* (CSDI_SOCI). In verifying the validity of CSDI_SOCI, we examine the relationship between CSDI_SOCI and corporate social performance indicators. To select social performance variables, we refer to the requirements of China Securities Regulatory Commission for listed firms to fulfil their corporate social responsibility. In this study, we follow Chen *et al*.’s definition of corporate social responsibility^70^ and select the following four indicators: disclosure of public relations and public welfare (*Public*), disclosure of the protection of suppliers’ rights and interests (*Suppliers*), disclosure of the protection of creditors’ rights and interests (*Creditor*), disclosure of the protection of customers and consumers’ rights and interests (*Custm_consm*). The validation results presented in Table 6. The testing results indicate that the CSDI_SOCI constructed in this study reflects the actual status of corporate CSR disclosure.

**Table 6:** Validation of CSDI_SOCI.

VARIABLES	*Public*	*Creditor*	*Supplier*	*Custm_consm*
*CSDI_SOCI*	9.050***	6.686***	4.731***	4.679***
	(16.70)	(14.28)	(9.84)	(9.65)
*Leverage*	−0.340**	−0.019	−0.538***	−0.241**
	(−2.55)	(−0.16)	(−4.38)	(−2.00)
*ROA*	1.452***	0.532	0.096	0.522
	(3.64)	(1.52)	(0.27)	(1.47)
*Share1*	−0.221	−0.256**	−0.143	0.039
	(−1.58)	(−2.05)	(−1.12)	(0.30)
*Big4*	0.580***	−0.329***	0.246***	0.435***
	(4.99)	(−4.01)	(3.05)	(4.70)
*Size*	0.668***	0.156***	0.492***	0.522***
	(28.01)	(8.06)	(24.09)	(25.06)
*Listage*	0.128***	0.189***	0.141***	0.135***
	(3.95)	(6.79)	(5.01)	(4.70)
*Constant*	−16.223***	−5.680***	−12.743***	−12.826***
	(−28.78)	(−12.42)	(−25.46)	(−25.53)
*Observations*	15,555	15,575	15,548	15,548
*Industry Fixed Effects*	YES	YES	YES	YES
*Year Fixed Effects*	YES	YES	YES	YES

Notes: ***p < 0.01, **p < 0.05, *p < 0.1; robust t-statistics in parentheses.

Last but more important, we test the validity of *Corporate Sustainability Disclosure Index* (CSDI). To measure the validity of CSDI, we construct aggregate corporate sustainability performance indicators and examine the relationship between CSDI and these indicators. First, we standardise the above-used twelve corporate sustainability performance indicators. Second, to ensure the robustness of the results, we adopt different weighting methods to assign weights to the twelve indicators and then calculate the composite sustainability indicator. The first composite indicator is obtained through an equally-weighted average of the standardised twelve sustainability indicators, denoted as *Score1*. The second composite indicator is calculated based on the application of the principal component analysis (PCA) method to the twelve sustainability indicators^71,72^, denoted as *Score2*. The third composite score is obtained based on the entropy method^73^, denoted as *Score3*. These validation results are presented in Table 7, respectively. The first columns in all three panels report the association of the composite score with CSDI, while the remaining three columns report that with the three dimension indexes (i.e., CSDI_ECO, CSDI_ENV, and CSDI_SOCI).

**Table 7:** Validation of CSDI.

VARIABLES	Panel A – *Score1*				Panel B – *Score2*				Panel C – *Score3*			
	*Score1*	*Score1*	*Score1*	*Score1*	*Score2*	*Score2*	*Score2*	*Score2*	*Score3*	*Score3*	*Score3*	*Score3*
*CSDI*	0.468***				1.066***				0.685***			
	(11.50)				(11.64)				(12.22)			
*Leverage*	−0.024	−0.015	−0.021	−0.008	−0.105*	−0.085	−0.105*	−0.074	−0.122***	−0.110***	−0.121***	−0.102***
	(−0.86)	(−0.53)	(−0.78)	(−0.30)	(−1.69)	(−1.36)	(−1.69)	(−1.19)	(−3.24)	(−2.88)	(−3.21)	(−2.68)
*ROA*	−0.021	−0.035	−0.037	−0.061	−0.255	−0.286	−0.287	−0.331*	−0.168	−0.186	−0.190*	−0.220*
	(−0.26)	(−0.43)	(−0.46)	(−0.75)	(−1.36)	(−1.51)	(−1.53)	(−1.76)	(−1.47)	(−1.62)	(−1.66)	(−1.92)
*Share1*	0.120***	0.117***	0.122***	0.115***	0.218***	0.211***	0.226***	0.210***	0.047	0.043	0.052	0.042
	(4.13)	(4.01)	(4.20)	(3.97)	(3.33)	(3.21)	(3.45)	(3.20)	(1.20)	(1.07)	(1.30)	(1.05)
*Big4*	0.079***	0.073***	0.078***	0.077***	0.183***	0.170***	0.183***	0.175***	0.066**	0.058**	0.065**	0.062**
	(3.93)	(3.65)	(3.89)	(3.84)	(3.87)	(3.60)	(3.88)	(3.70)	(2.38)	(2.08)	(2.37)	(2.22)
*Size*	0.125***	0.126***	0.124***	0.130***	0.289***	0.291***	0.284***	0.295***	0.149***	0.151***	0.146***	0.154***
	(25.98)	(26.06)	(25.56)	(26.64)	(25.91)	(25.97)	(25.42)	(26.24)	(22.44)	(22.52)	(21.95)	(22.93)
*Listage*	0.015**	0.013*	0.014**	0.015**	−0.008	−0.013	−0.012	−0.012	0.005	0.002	0.003	0.003
	(2.27)	(1.94)	(2.00)	(2.14)	(−0.53)	(−0.83)	(−0.76)	(−0.77)	(0.58)	(0.25)	(0.32)	(0.35)
*CSDI_ECO*		0.247***				0.609***				0.428***		
		(2.72)				(2.98)				(3.42)		
*CSDI_ENV*			0.500***				1.408***				0.840***	
			(8.85)				(10.90)				(10.67)	
*CSDI_SOCI*				1.004***				1.201***				0.974***
				(9.62)				(5.12)				(6.85)
*Constant*	−3.148***	−3.087***	−3.026***	−3.153***	−7.063***	−6.932***	−6.773***	−6.965***	−2.945***	−2.866***	−2.762***	−2.903***
	(−27.89)	(−27.01)	(−26.83)	(−27.87)	(−27.57)	(−26.68)	(−26.48)	(−27.04)	(−19.28)	(−18.48)	(−18.09)	(−18.92)
*Observations*	12,672	12,672	12,672	12,672	12,672	12,672	12,672	12,672	12,672	12,672	12,672	12,672
*R-squared*	0.206	0.197	0.202	0.202	0.209	0.201	0.209	0.202	0.186	0.177	0.185	0.179
*Industry Fixed Effects*	YES	YES	YES	YES	YES	YES	YES	YES	YES	YES	YES	YES
*Year Fixed Effects*	YES	YES	YES	YES	YES	YES	YES	YES	YES	YES	YES	YES

Notes: ***p < 0.01, **p < 0.05, *p < 0.1; robust t-statistics in parentheses; *Score1:* Composite sustainability score calculated based on equal-weighting scheme; *Score2:* Composite sustainability score calculated based on the principal component analysis (PCA); *Score3:* Composite sustainability score calculated based on the entropy method.

Overall, the associations between CSDI and all composite corporate sustainability scores are significantly correlated. Similar results are reached for the three dimension scores. Accordingly, the validation results document that the CSDI constructed in this study can reflect the actual performance of firms’ sustainable development.

Taken together, these validation results provide solid evidence that the constructed *Corporate Sustainability Disclosure Index* (CSDI), *CSDI_Economic Dimension* (CSDI_ECO), *CSDI_Environmental Dimension* (CSDI_ENV), and *CSDI_Social Dimension* (CSDI_SOCI) are valid and can reflect the actual disclosure of corporate sustainability performance.

### Limitations

Our dataset has the following limitations. First, limited by the availability of latest firm-level data, the dataset constructed in this study does not take into account the impact of the COVID-19 pandemic on corporate sustainability. Second, comparisons between different industries are beyond the scope of current study and can be further explored in future studies. Third, the sustainability information disclosure index constructed in this study does not fully consider the context of the words. Although negative tones are very rare in the MD&A documents, a lack of context consideration might still generate some (albeit very limited) influences^25,29^. Future studies can make attempts to improve machine learning techniques to take into account the context where key words are located when constructing text-based quantitative measures. Lastly, as market conditions in developed countries are different from those in developing countries such as China, future studies can build on the spirit of this study to investigate the progress of corporate sustainability disclosure in developed countries.

## Data Availability

The codes used for calculation and analysis in this study are available in *figshare*^74–78^.
